# Welfare State Regimes, Gender, and Depression: A Multilevel Analysis of Middle and High Income Countries

**DOI:** 10.3390/ijerph10041324

**Published:** 2013-03-28

**Authors:** Haejoo Chung, Edwin Ng, Selahadin Ibrahim, Björn Karlsson, Joan Benach, Albert Espelt, Carles Muntaner

**Affiliations:** 1 Department of Health Care Management, Korea University, Seoul 136-703, Korea; E-Mail: hpolicy@korea.ac.kr; 2 Dalla Lana School of Public Health, University of Toronto, Toronto, ON M5T 1P8, Canada; E-Mails: edwin.ng@utoronto.ca (E.N.); sibrahim@iwh.on.ca (S.I.); 3 Institute for Work & Health, Toronto, ON M5G 2E9, Canada; 4 Department of Psychiatry and Neurochemistry, Institute of Neuroscience and Physiology, Sahlgrenska Academy, University of Gothenburg, Gothenburg 405 30, Sweden; E-Mail: karlsson.bjorn@gmail.com; 5 Health Inequalities Research Group (GREDS), Employment Conditions Network (EMCONET), University of Pompeu Fabra, Barcelona 08003, Spain; E-Mail: joan.benach@upf.edu; 6 Public Health Agency of Barcelona (ASPB), Barcelona 08023, Spain; E-Mail: aespelt@aspb.cat; 7 CIBER of Epidemiology and Public Health (CIBERESP), Barcelona 08036, Spain; 8 Bloomberg Faculty of Nursing, University of Toronto, Toronto, ON M5T 1P8, Canada

**Keywords:** welfare state regime, multilevel, global mental health, depression, gender

## Abstract

Using the 2002 World Health Survey, we examine the association between welfare state regimes, gender and mental health among 26 countries classified into seven distinct regimes: Conservative, Southeast Asian, Eastern European, Latin American, Liberal, Southern/Ex-dictatorship, and Social Democratic. A two-level hierarchical model found that the odds of experiencing a brief depressive episode in the last 12 months was significantly higher for Southern/Ex- dictatorship countries than for Southeast Asian (odds ratio (OR) = 0.12, 95% confidence interval (CI) 0.05–0.27) and Eastern European (OR = 0.36, 95% CI 0.22–0.58) regimes after controlling for gender, age, education, marital status, and economic development. In adjusted interaction models, compared to Southern/Ex-dictatorship males (reference category), the odds ratios of depression were significantly *lower* among Southeast Asian males (OR = 0.16, 95% CI 0.08–0.34) and females (OR = 0.23, 95% CI 0.10–0.53) and Eastern European males (OR = 0.41, 95% CI 0.26–0.63) and significantly higher among females in Liberal (OR = 2.00, 95% CI 1.14–3.49) and Southern (OR = 2.42, 95% CI 1.86–3.15) regimes. Our results highlight the importance of incorporating middle-income countries into comparative welfare regime research and testing for interactions between welfare regimes and gender on mental health.

## 1. Introduction

The last decade of the 20th century saw an expansion of studies on the macrosocial determinants of population health [1] mostly focused on economic (income inequality [2]) and cultural (social capital [3]) factors. By the end of the century some researchers noted the lack of consideration of political factors in these models [4]. Soon after, a new research program emerged on the macro comparative politics of population health [5,6,7,8,9,10,11,12,13] centered on the construct of welfare state regimes [14]. 

The state role as provider for the welfare of its population grew in Europe during the 20th century and found a major boost in its second half. The development of European welfare states is characterized by the political conviction that providing goods and services to the population is a matter of citizenship rights and not of philanthropy or charity [14]. The associated term of welfare regime [15] integrates the traditional provision of goods and services by the state with its labor market policies. 

To date, most comparative epidemiological studies have used ecological designs because of a lack of available data. Lack of individual level datasets with political variables and, most critically, lack of multilevel datasets at the national level partially accounts for this limitation. Multilevel analyses first appeared in social epidemiology during the mid-nineties [16,17]; however, these studies were typically conducted at local or regional levels. Furthermore, the relatively strong development of welfare states in Europe, North America, and Australia explain why theoretical models for comparative welfare regime analysis have been produced mostly in Europe and North America [14,15,18,19]. For example, Eikemo and colleagues [7] examined welfare state variations in self- reported health, and found that Southern European countries had worse self-reported health than the Social Democratic and Liberal welfare regime types. The structural weakness of the weak welfare state and economic inequalities stemming from dictatorships in Spain, Greece and Portugal can explain the relatively worst performance of this welfare regime type [20]. 

Conversely, there are no global welfare regimes typologies or well-developed overarching conceptual frameworks to account for welfare state variation beyond Organization for Economic Co-operation and Development (OECD) countries [21]. One such candidate could be Gough and Wood’s classification of welfare regimes in Latin America, Asia and Africa [22,23]. A problem with such typology is that it is based on the United Nation’s Human Development Index and thus conflates origins and outcomes of welfare regimes, including health. As a result, several authors have conducted comparative analyses of Southeast Asian, former Communist Soviet countries (e.g., Eastern Europe), and Latin America which show that the welfare regimes of these regions tend to share some common features that justify grouping them together in distinct clusters [24,25]. Table 1 lists four key dimensions of welfare regimes that might influence population levels of mental health, and compares seven global welfare regimes across these dimensions. First, population coverage refers to the share of the population eligible for welfare services and benefits based on social citizenship. Welfare regimes range from universal population coverage (e.g., welfare benefits are available to all citizens and are designed to promote and equalize social and economic opportunities) and occupational (e.g., welfare benefits are often tied to the past employment of male breadwinners) to selective coverage (e.g., benefits are restricted to those without sufficient financial means) [26]. It follows that mental health outcomes should be more favorable across and within national contexts where coverage is universal because the egalitarian principles are institutionalized. Second, the role of the private market reflects the degree to which the private sector is involved with meeting basic needs and providing welfare services. Among welfare regimes where the market and private schemes play central roles, individuals in need are eligible for only minimal supports, and consequently, are more likely to experience mental health problems compared to those who can afford private services.

**Table 1:** General features of welfare state regimes.

Regime	Population Coverage ^1^	Role of the Private Market ^2^	Target Population ^3^	Decommodification ^4^
Social Democratic	Universal	Low	All citizens	High
Conservative	Occupational	Low	Families	Medium
Southern/Ex-dictatorship	Occupational	Medium	Families	Low
Liberal	Selective	High	Poor	Low
Eastern European	Selective	Medium	Poor	Low
Southeast Asian	Selective	Low	Families	Very low
Latin American	Occupational	High	Families	Very low

*Sources*: Esping-Andersen [14]; Ferrera [19]; Ramesh [27]; Haggard and Kaufman [24]; *Notes*: ^1^ Population Coverage = share of the population eligible or covered for welfare services and benefits; ^2^ Role of Private Market = degree to which welfare needs are met through the private sector; ^3^ Target Population = individuals or groups that are identified as the primary and intended recipients of welfare services and benefits; ^4^ Decommodification = degree to which welfare benefits reduce individuals’ reliance on the market to meet basic needs.

Third, welfare regimes tend to deliver services and benefits to target populations who are the intended recipients of welfare efforts. Whether a welfare regime targets the needs of its entire citizenry, families, or simply the most marginalized reflects the relative roles that state, market, and families are expected to play in meeting welfare needs. For example, women in welfare states that implement comprehensive policies for all citizens are less dependent on the market (e.g., employment wages) or their families (e.g., household income) or both to secure an acceptable standard of living [28]. In countries where this process of defamilization occurs, women should experience favorable mental health outcomes compared to less independent women in regimes that primarily target families or the poor. Another important dimension that differentiates welfare regimes is Esping-Andersen’s [14] index of decommodification, which refers to the extent to which unemployment, sickness, and pension benefits reduce an individual’s reliance on the labor market to meet basic needs. This dimension is closely related to the concept of redistribution, or degree to which countries narrow social inequalities through social transfers. Past welfare regime research has found that as levels of decommodification and redistribution increase, so do indicators of population health [29,30].

Comparing these dimensions across specific welfare regimes reveals that Social Democratic countries such as Sweden and Norway form a distinct regime and are uniquely egalitarian through the universal and generous provision of welfare benefits to all citizens. In contrast, the Conservative regime, which includes Germany and France, tends to deliver welfare programs based on a social insurance model for male wage earners and families while Liberal nations like Australia, the US, and UK emphasize free market and individualistic solutions such as means-testing to determine eligibility for welfare benefits. Ferrera [19] added a fourth European regime which included Southern welfare states such as Greece, Portugal, and Spain. The welfare regimes of these Southern countries tend to be highly fragmented and corporatist in nature with minimal state involvement in welfare affairs, low levels of decommodification, and high levels of “clientelism” (e.g., exchanging valuable goods and services for political capital and support). To date, social epidemiology research has heavily relied on Esping-Andersen’s typology to examine the link between comparative politics and population health, reaching the overall conclusion that more generous welfare states (Social Democratic and Conservative countries) tend to be associated with better average health indicators than less generous welfare states (e.g., Liberal and Southern nations [31,32]).

Regarding low- and middle-income regimes, recent evidence on labor market characteristics supports the notion that East European, Southeast Asian, and Latin American countries do cluster into distinct welfare regimes [33,34]. For example, middle-income Eastern European welfare states are characterized by a “social/liberal” mix where a state in retrenchment still controls a weak welfare state in the midst of economic liberalization [35]. The institutional structure and welfare generosity of these countries tends to be more fragmented, restrictive, and less generous compared to their European counterparts. As a result, these countries rely heavily on families and charities to augment the financing and delivery of welfare services. Southeast Asian middle-income countries, on the other hand, can be characterized by weak welfare state provisions in the context of substantial informality [24]. These countries are often considered emerging and productivist, reflecting their primary commitment to economic productivity and growth and secondary interest to the provision of essential welfare services. These regimes are based on a minimal level of state involvement, selective and gradual expansion of welfare coverage, and a heavy reliance on non-state actors such as firms, families, and communities to provide welfare services. As for Latin American countries, these middle-income nations are also considered developing welfare states and have experienced a surge in social expenditures including health and child development (e.g., Oportunidades program in Mexico and Bolsa da Familia in Brazil) in the middle of high income inequality and labor market informality [36].

Clearly, the scholarly need to map the relation between welfare regimes and population mental health beyond OECD nations exists and to use multilevel methods to account macro- and micro-level factors. In this paper, our goal is to advance the field of comparative social epidemiology of welfare regimes and population health by adding Southeast Asian, Latin American and Eastern European semi-institutionalized welfare regime types to the Nordic, Continental, Anglo Saxon and Southern European types that have dominated the literature of welfare regime analyses [37]. A second goal is to provide inferences at the individual level using multilevel methods that capture welfare regimes at the country level. The individual level outcome is depression, a common mental disorder that significantly contributes to the global burden of disease [38], and varies significantly across global regions [39]. A multilevel analysis allows us to examine specific health inequality generating mechanisms via the testing of cross-level interactions involving welfare state regimes as a macro-social determinant. That is, welfare regimes would modify the association of individual level attributes. Another important determinant of health that might be modified by welfare state regime is gender [40]. More specifically, Nordic welfare state regimes are known to have achieved greater gender equity while patriarchal welfare regimes such as those in southern Europe tend to lag in gender equity [20]. Compared to traditional risk factors such as poor diet or smoking, the implications of welfare states for health are not straightforward. Mental health outcomes appear to be sensitive to gender dynamics and welfare state regimes through their combined impact on poverty [41], a major risk factor for depression among women [42]. 

Given these contributions, we use multilevel methods to analyze inequalities in depression across a wide range of welfare regimes including Nordic (Social Democratic), Continental (Conservative), Southern (Southern/Ex-dictatorship), Anglo-Saxon (Liberal), Eastern European, Southeast Asian and Latin American, and to examine possible cross-level interactions between gender and welfare state regimes. Based on the existing research on comparative regimes, gender, and health [43], we anticipate that: (i) survey respondents who are women, older, less “credentialed”, and not married will experience higher rates of depression; (ii) Conservative, Liberal, Social Democratic, and Eastern European welfare regimes will fare better compared to the Southern/Ex-dictatorship regime; and (iii) gender will significantly interact with welfare regimes in relation to depression. Given that past research has not explored the impact on Southeast Asian and Latin American regimes and mental health, we offer no tentative hypotheses.

## 2. Methods

We analyzed cross-sectional data derived from the 2002–2005 World Health Survey (WHS) conducted by the World Health Organization (WHO) [44,45,46]. Using a multi-stage, stratified cluster sampling procedure, the WHO conducted national surveys in 70 low-, middle-, and high-income countries among adults aged 18 years and older, generating nationally comparable and representative samples for population health assessment. With the exception of a few countries not included in the current analyses, response rates were high at both individual and household levels (ranging from 70% to 100%). Sampling weights were modified with post stratification and applied for non-responses. We selected national samples from countries representing Nordic, Continental, Anglo-Saxon, Latin American, Southeast Asian, and Southern and Eastern European countries. Specifically, the following 26 middle- and high-income countries were selected: Australia (AUT), Austria (AUS), Belgium (BEL), Brazil (BRA), Czech Republic (CZE), Denmark (DNK), Estonia (EST), Finland (FIN), France (FRA), Germany (DEU), Great Britain (GBR), Greece (GRC), Hungary (HUN), Ireland (IRL), Italy (ITA), Malaysia (MYS), Mexico (MEX), Netherlands (NLD), Norway (NOR), Philippines (PHL), Portugal (PRT), Slovakia (SVK), Slovenia (SVN), Spain (ESP), Sweden (SWE), and Uruguay (URY). Limiting our sample to these countries yielded 93,505 possible participants aged 18 and older. These particular countries were selected on theoretical and methodological grounds. Theoretically, we are interested in augmenting contemporary welfare regime theory by testing two emerging and unexplored welfare regimes: Southeast Asian and Latin American. The inclusion of these two regimes expands upon the theoretical scope of Esping-Andersen’s original typology, and the recent work conducted by Eikemo *et al.* [7,8] on Southern and Eastern European countries. In terms of methods, our sampling frame was limited to countries that participated in the WHS. We selected every country that was available in the WHS that met our inclusion criteria; however, a large number of non-participating countries reduced our overall sample size, including Southeast Asian countries (e.g., Indonesia, Vietnam, and Thailand), and Latin American nations (e.g., Columbia, Argentina, and Venezuela). 

### 2.1. Variables

#### 2.1.1. Dependent Variable

*Brief depressive episode*. Depression is measured with the International Classification of Diseases Tenth Edition’s (ICD-10) criteria for brief depressive episode. During the last 12 months, participants had to report “yes” to two out of three following questions: (i) “Have you had a period lasting several days when you felt sad, empty or depressed?”; (ii) “Have you had a period lasting several days when you lost interest in most things you usually enjoy such as hobbies, personal relationships or work?”, or (iii) “Have you had a period lasting several days when you have been feeling your energy decreased or that you are tired all the time?” Furthermore, participants had to answer “yes” to the following two questions: “Change in appetite/weight loss” and “Diminished ability to think or concentrate”. Participants answering “yes” to at least four symptoms were classified as experiencing a brief depressive episode.

#### 2.1.2. Independent Variables

*Welfare state regimes*. Twenty-six countries were coded into seven distinct welfare regimes based on the typologies developed by Esping-Andersen [14], and later expanded by Ferrera [19,47], Haggard and Kaufman [24], and Ramesh [27]: (i) Conservative (n = 6: AUT, BEL, DEU, NLD, FRA, ITA); (ii) Liberal (n = 3: AUS, GBR, IRL); (iii) Social Democratic (n = 4: DNK, FIN, NOR, SWE); (iv) Southeast Asian (n = 2: MYS, PHL); (v) Eastern European (n = 5: CZE, EST, HUN, SVK, SVN); (vi) Latin (n = 3: BRA, MEX, URY); and (vii) Southern/Ex-dictatorship (n = 3: ESP, GRC, PRT). The last regime served as the reference category. To account for country differences in economic development, GDP per capita (current US$) and GDP annual growth rate (%) data were retrieved from the World Bank and measured as three-year averages (2002–2005) [48,49].

*Socio-demographics.* *Gender* was coded as female and male (reference category). *Age* was operationalized into six categories: 18–29 (reference category), 30–39, 40–49, 50–59, 60–69, and 70+. *Educational attainment* was measured in four categories: less than primary school (reference category), primary school completed, secondary school completed, and post-secondary/graduate school completed. *Marital status* was measured as never married (reference category), married/cohabiting, and divorced/separated /widowed).

### 2.2. Analysis

Multilevel logistic regressions were used to assess the associations between depression, gender and welfare state regime while taking into account age, educational attainment, and marital status. Analyses involved measuring individuals nested within countries at Level 1 and conceptualizing countries at Level 2. A two-level random effects model was constructed to explain depression variability among individuals by introducing welfare regime as a fixed effects indicator at the country-level. Of the possible respondents, 98.5% of participants had no missing values (n = 92,060). Statistical analyses included bivariate, multivariate, and interaction models between welfare regime and gender on depression. Intra-class correlations (ICC) were conducted to ascertain the total variance in depression that might be attributable to between-country variation. Sensitivity tests were also used to exclude welfare regimes in stepwise fashion to evaluate the stability of our final results. SAS and MPlus7 software were used in the analyses.

## 3. Results

**Figure 1:**
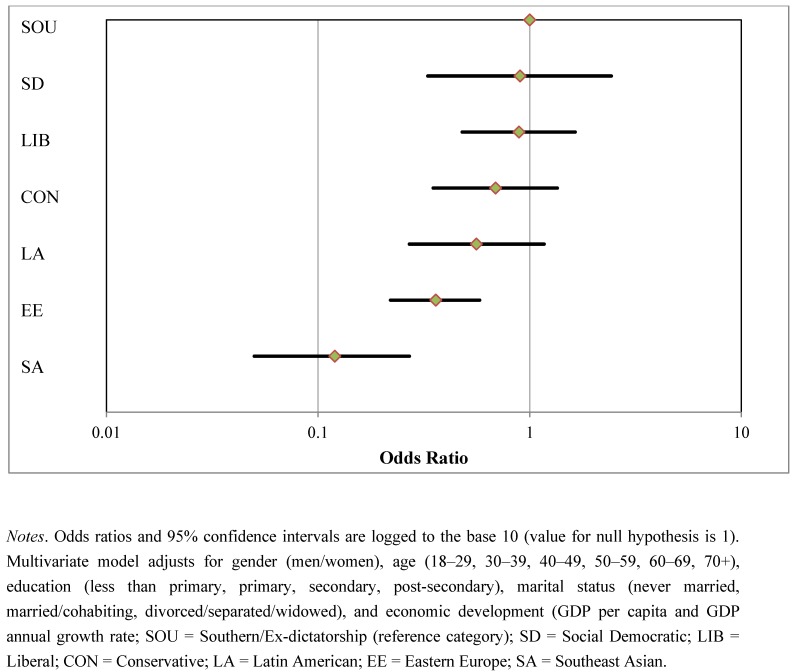
Odds ratios with 95% confidence intervals on the multivariate effect of welfare state regimes on depression.

Descriptive statistics for our variables are presented in Table 2. Model 1 in Table 3 shows bivariate regressions for brief depression episode on all predicator variables. As hypothesized, women, older persons, those with less than primary education, and those who were divorced, separated or widowed at time of the interview were significantly more likely to have experienced a brief depressive episode. The associations between depression with age and education followed a “dose-response” pattern—as individuals aged and acquired more education, depressive episodes increased and decreased, respectively. Other global surveys such as the World Mental Health Survey have shown slightly different patterns for specific periods and locations; however, these results are quite consistent with most data in middle- and high-income countries with respect to age, education, marital status, and gender [50]. We found partial support for our second hypothesis, associations between welfare regimes and depression found that participants from Southeast Asia (odds ratio (OR) = 0.17, 95% confidence interval (CI) 0.08–0.36), Eastern European (OR = 0.39, 95% CI 0.21–0.73), Social Democratic (OR = 0.43, 95% CI 0.22–0.84), and Conservative (OR = 0.45, 95% CI 0.24–0.82) regimes fared significantly better compared to those from the Southern/Ex-dictatorship regime (reference category). No significant differences were found between Liberal and Latin American regimes and the reference category. Model 2 controls for individual- and country-level variables simultaneously in a single multilevel logistic regression. Adjusted results indicate that only Southeast Asian (OR = 0.12, 95% CI 0.05–0.27) and Eastern European (OR = 0.36, 95% CI 0.22–0.58) regimes remained statistically significant (lower odds of depression) compared to Southern/Ex-dictatorship countries (see Figure 1). 

**Table 2:** Sample description.

Variable	Frequency	Percent ^a^
*Brief Depressive Episode*		
Yes	5,422	6
No	86,638	94
*Gender*		
Men	52,645	57
Women	39,415	43
*Age*		
18–29	23,146	25
30–39	21,003	23
40–49	16,696	18
50–59	12,451	14
60–69	9,761	10
70–120	9,003	10
*Marital Status*		
Never Married	17,798	19
Married/Cohabiting	59,747	65
Divorced/separated/widowed	14,515	16
*Educational Attainment*		
Less than Primary	7,188	8
Primary	16,720	18
Secondary	59,529	65
Post-secondary/Graduate	8,623	9
*Welfare State Regime*		
Conservative	5,595	6
Liberal	5,570	6
Social Democratic	3,974	4
Southeast Asian	16,105	18
Eastern European	5,701	6
Latin American	46,723	51
Southern/Ex-dictatorship	8,392	9

*Notes.* n = 92,060; ^a^ Percentage values are rounded.

**Table 3:** ORs and 95% CIs for bivariate, multivariate, and interactive regressions.

Level/Variable	Bivariate (Model 1)	Multivariate (Model 2)	Interaction (Model 3)
	OR (95% CI)	OR (95% CI)	OR (95% CI)
**Level 1: Individual Level**			
*Gender*			
Men	1 (reference)	1 (reference)	
Women	2.15 (2.02–2.28)	1.99 (1.82–2.17)	
*Age*			
18–29	1 (reference)	1 (reference)	1 (reference)
30–39	1.40 (1.27–1.53)	1.42 (1.28–1.57)	1.42 (1.29–1.57)
40–49	1.81(1.65–1.99)	1.76 (1.59–1.95)	1.76 (1.59–1.96)
50–59	2.18 (1.98–2.40)	1.94 (1.50–2.51)	1.94 (1.50–2.52)
60–69	2.20 (1.98–2.44)	1.77 (1.36–2.31)	1.78 (1.37–2.32)
70+	2.43 (2.20–2.70)	1.67 (1.04–2.66)	1.67 (1.05–2.67)
*Educational Attainment*			
Primary	0.82 (0.74–0.91)	0.89 (0.71–1.12)	0.89 (0.71–1.12)
Secondary	0.50 (0.45–0.56)	0.66 (0.53–0.82)	0.66 (0.53–0.82)
Post-secondary	0.40 (0.34–0.46)	0.51 (0.40–0.65)	0.52 (0.41–0.66)
*Marital Status*			
Never married	1 (reference)	1 (reference)	1 (reference)
Married/cohabiting	1.12 (1.04–1.22)	0.83 (0.71–0.97)	0.83 (0.71–0.97)
Div/sep/wid	2.45 (2.25–2.68)	1.39 (1.31–1.48)	1.39 (1.30–1.48)
**Level 2: Country Level**			
*Welfare State Regime*			
Southern/Ex-Dict. (SOU)	1 (reference)	1 (reference)	
Conservative (CON)	0.45 (0.24–0.82)	0.69 (0.35–1.35)	
Liberal (LIB)	0.55 (0.27–1.11)	0.89 (0.48–1.64)	
Social Democratic (SD)	0.43 (0.22–0.84)	0.90 (0.33–2.43)	
Southeast Asian (SA)	0.17 (0.08–0.36)	0.12 (0.05–0.27)	
Eastern European (EE)	0.39 (0.21–0.73)	0.36 (0.22–0.58)	
Latin American (LA)	0.66 (0.33–1.31)	0.56 (0.27–1.17)	
*Male X Welfare State*			
Male X SOU			1 (reference)
Male X CON			0.83 (0.43–1.63)
Male X LIB			1.19 (0.66–2.12)
Male X SD			1.48 (0.62–3.54)
Male X SA			0.16 (0.08–0.34)
Male X EE			0.41 (0.26–0.63)
Male X LA			0.62 (0.31–1.23)
*Female X Welfare State*			
Female X SOU			2.42 (1.86–3.15)
Female X CON			1.59 (0.88–2.88)
Female X LIB			2.00 (1.14–3.49)
Female X SD			1.77 (0.57–5.46)
Female X SA			0.23 (0.10–0.53)
Female X EE			0.80 (0.51–1.26)
Female X LA			1.30 (0.66–2.56)
ICC	9.6% ^a^	4.5% ^b^	4.5% ^c^
Intercept		1.095	1.304
Variance		0.043	0.042
−2logL		−19,269.422	−19,255.427
*N*		92,060	92,060

***Notes*.** Model 1 runs separate models for each explanatory variable; Models 2 and 3 also adjust for GDP per capita (continuous) and GDP annual growth rate (continuous) (not shown). In both models, economic development variables were insignificant at <0.05; Div/sep/wid = divorced/separated/widowed; ICC = intra-class correlation coefficient, represents the change in country-level variance compared to the intercept-only model’s ICC (10.0%); ^a^ ICC includes only individual-level covariates; ^b^ ICC includes all individual-level covariates and country-level welfare regimes; ^c^ ICC includes all individual-level covariates and interactions between welfare regimes and gender.

In Model 3, the interactive effects between welfare regimes and gender on depression were tested while controlling for socio-demographic and economic development factors. As compared with males in Southern/Ex-dictatorship nations (reference category), Southeast Asian males (OR = 0.16, 95% CI 0.08–0.34) and females (OR = 0.23, 95% CI 0.10–0.53) and Eastern European males (OR = 0.41, 95% CI 0.26–0.63) had lower odds of experiencing a brief depressive episode in the past year. Females from Liberal (OR = 2.00, 95% CI 1.14–3.49) and Southern/Ex-dictatorship (OR = 2.42, 95% CI 1.86–3.15) regimes, on the other hand, had significantly higher odds of experiencing depression than the reference category. Interactions between gender and the remaining regimes were non-significant. ICC results found that the intercept-only model explained 10.1% of the total outcome variance due to between-welfare regime differences. After adding individual- and country-level variables, the ICCs were reduced to 9.6% and 4.9%, respectively. In other words, nearly 95% of the variation in brief depressive episodes were at the individual-level while welfare regime characteristics accounted for the remaining of 5%. Sensitivity tests revealed that excluding individual welfare regimes in stepwise fashion from interaction models did not substantially change our central findings. However, the odds ratio for females from Conservative (OR = 2.28, 95% CI 1.28–4.08) countries did reach significance when the Liberal regime was excluded from analysis (see model 3 in Appendix Table A1).

## 4. Discussion

Our expanded analysis on the associations between welfare regimes, gender, and mental health beyond wealthy countries corroborates the findings of previous work and offers novel findings. On one hand, this study’s findings are consistent with other comparative research in confirming that Southern/Ex-dictatorship regimes tend to experience the highest rates of mental health problems among European welfare states [7], and that the association between gender and morbidity varies across welfare regimes [10]. This study augments the extant literature on comparative regimes and population health in three important ways. First, this study uses ICD-10 diagnostic criterion to measure brief depressive disorder among middle- and high-income welfare regimes [51]. By using the WHO’s standard diagnostic tool, our measurement of brief depression represents a significant improvement over past research that have relied on self-report questions. Advantages of the ICD-10 include its diagnostic ability to capture the cultural and ethnic contexts of brief depression, validation and reliability of translated versions, and wide-acceptance as the international standard to research mental disorders. Second, this study conceptualized ten middle-income countries into three distinct welfare regimes: Latin American, Eastern European, and Southeast Asian. The two latter regimes, in fact, were found to have lower odds of experiencing depression compared to the Southern/Ex-dictatorship regime even after controlling for individual- and country-level factors. Given that most comparative research has focused exclusively on wealthy countries in Europe, North America, and Australia, the inclusion of non-OECD countries advances our understanding of emerging welfare states and complements the burgeoning literature on global health. Third, this study heeds Bambra and colleagues’ [10] call to engage more with issues of gender across regimes, and found that the association between welfare regimes and depression was modified by gender. Compared to the reference category (Southern/Ex-dictatorship males), males in Southeast Asian (OR = 0.16) and Eastern European (OR = 0.41) countries fared significantly better as well as Southeast Asian females (OR = 0.23). Conversely, mental health disadvantages were observed among females from Liberal (OR = 2.00) and Southern (OR = 2.42) regimes.

There are several possible explanations for these associations. The mental health advantages seen among males and females in Southeast Asian nations are somewhat surprising given that these countries are characterized by residual or developmental welfare states. Although these welfare regimes are less likely to intervene in market-generated inequalities, invest in social welfare, or implement publicly-funded protection systems, Southeast Asian nations still fared better than more generous and established welfare states. This counterintuitive finding suggests that non-welfare regime characteristics such as social, economic, or cultural determinants may be more influential in affecting population levels of mental health. For example, research on the global epidemiology of depression finds a marked contrast between wealthy and non-wealthy regions of the world. The burden of depressive disorders is more than twice as high in high-income nations (8.9%) compared to the burden experienced by low- and middle-income countries (4.1%) [52]. In terms of cultural factors, comparative psychiatry has long shown that cultural norms, values, and expectations influence the symptom profiles, courses, and outcomes of various mental health disorders. Whereas depression is widely accepted as a psychiatric syndrome in Europe and North America, there is some debate on the equivalence of such a syndrome in some traditional Southeast Asian cultures [53]. These considerations point to the need for more work among Southeast Asian nations to better understand the gendered experience and the cultural distribution of depression.

According to welfare regime theory, the higher odds of brief depression among women in Liberal and Southern/Ex-dictatorship countries might be due to the *subordinate role* that these respective states play in promoting women’s “access to paid work” [54], providing welfare services, and supporting policies that “decommodify” women’s labor [14]. First, Liberal and Southern regimes are characterized by under-developed family policies that fail to support mothers in their dual roles as caretakers and workers (e.g., unpaid maternity leaves, short leaves, meager and strict benefits). Without extensive childcare provisions, women bear a disproportionate share of childcare duties and have limited access to paid work, both of which contribute to elevated risks of mental health disorders. Family-friendly policies at the welfare regime level are closely related to the idea of defamilization, or the degree to which women can remain economically independent from traditional “male breadwinner” models [55]. In this sense, Liberal and Southern nations do little to decommodify the role of women in families. Second, the socioeconomic position of women in these regimes tends to be disadvantaged since these states are non-interventionist in providing essential welfare services (e.g., early childhood education, healthcare, housing supports) and in promoting gender quality over the life-course (e.g., affirmative action policies, equal pay for equal work, quotas for female participation in governing bodies). In place of an interventionist state, the market assumes a dominant role in subsidizing private welfare schemes [14], leaving women more susceptible to poor material conditions and psychosocial stressors. Third, the extent to which women can secure an acceptable standard of living, independent of market performance or familial supports, in Liberal and Southern welfare states is comparatively low. Hence, the social protections available for women in these countries tend be strict (e.g., benefits are available for a small segment of the population), minimal (e.g., social assistance benefits remain below poverty thresholds), and stigmatizing (e.g., benefits are viewed negatively from greater society). For example, income redistribution schemes such as unemployment benefits are essential to buffering market generated inequalities and ensuring the economic security of women and families. However, since these protections tend to be means-tested, the amount of income transferred is limited, and the stigma of receiving such benefits is high. Taken together, these proposed mechanisms make clear that Liberal and Southern/Ex-dictatorship regimes mediate the extent, and impact, of mental health outcomes among women. More theoretical work, however, is needed to trace the mechanisms that explain which welfare state contexts (e.g., social insurance models) interact with vulnerable females (e.g., single mothers) to produce avoidable and treatable mental health outcomes.

A number of caveats need to be considered. First, a critical realist perspective of our measure of depression would demand a deeper examination of its assumed validity across various cultural contexts [56]. Further work should integrate critical realism and comparative psychiatry to produce a more nuanced and contextual understanding on the social mechanisms that link welfare regimes and mental health. Second, our categorization of the Southeast Asian regime only consisted of Malaysia and Philippines since other countries such as Indonesia, Laos, Thailand, or Vietnam did not participate in the WHS. A similar critique applies to our Liberal cluster given that the US and Canada were not available for inclusion. Despite this drawback, our sensitivity tests did not find evidence for selection bias when the Southeast Asian and Liberal regimes were excluded. A second potential limitation involves our regime approach to conceptualizing welfare states [14]. Although a regime approach allowed us to consider the interconnected associations between social structures, welfare institutions, and population health, it also hindered our ability to test specific pathways and mechanisms. Alternatively, welfare states can also be conceptualized using government expenditures (e.g., social or education spending as a percentage of GDP) [57]. Since the regime (*i.e.*, levels of decommodification) and welfare effort approaches (*i.e.*, measures of social spending) are strongly associated (e.g., Social Democratic nations are also the most generous), we doubt our findings would substantially change if the latter approach was used. Third, it is possible that reverse causation might account for this study’s findings (e.g., the probability of depression is causally related to exposures associated with welfare regimes). The WHS was a cross-sectional survey, did not include questions on onset or duration of depressive episodes, and as a result, we are unable to differentiate between cause and effect. With this said, retrocausality appears to be a more salient explanation for outcomes among low-income countries rather than middle- or high-income nations [58]. 

## 5. Conclusions

In sum, our multilevel study of brief depression demonstrates the value of expanding beyond high-income countries to include emerging welfare regimes from middle-income countries. A central finding is that the least generous form of welfare regime type, namely the Southern/Ex-dictatorship type was more strongly associated with depression than middle-income nations in Southeast Asia and Eastern European. Future research should expand the number of middle-income countries and probe for the stability of the reported findings. Given the depth of the current recession in Southern/Ex-dictatorship countries and the structural weakness of their welfare regimes [59], it is likely the present findings will be even amplified in future studies. 

## Appendix

**Table A1:** Sensitivity analyses, stepwise exclusion of individual welfare regimes in interactive models.

Variation	Model 1 OR (95% CI)	Model 2 OR (95% CI)	Model 3 OR (95% CI)	Model 4 OR (95% CI)	Model 5 OR (95% CI)	Model 6 OR (95% CI)
*Male X* *Welfare State*						
Male X SOU	1 (reference)	1 (reference)	1 (reference)	1 (reference)	1 (reference)	1 (reference)
Male X CON	-	0.75 (0.40–1.42)	1.20 (0.63–2.30)	0.83 (0.43–1.62)	0.82 (0.38–1.74)	0.77 (0.40–1.49)
Male X LIB	1.30 (0.74–2.30)	-	1.82 (0.91–3.62)	1.15 (0.64–2.05)	1.21 (0.66–2.18)	1.29 (0.74–2.27)
Male X SD	1.65 (0.69–3.95)	1.04 (0.45–2.41)	-	1.43 (0.59–3.46)	1.49 (0.60–3.70)	1.51 (0.66–3.44)
Male X SA	**0.15 (0.07–0.32)**	**0.19 (0.09–0.39)**	**0.10 (0.04–0.25)**	**-**	**0.17 (0.06–0.46)**	**0.18 (0.08–0.38)**
Male X EE	**0.40 (0.26–0.61)**	**0.42 (0.28–0.62)**	**0.31 (0.17–0.56)**	**0.40 (0.26–0.62)**	**-**	**0.46 (0.30–0.71)**
Male X LA	0.56 (0.29–1.12)	0.79 (0.41–1.52)	0.42 (0.19–0.93)	0.64 (0.32–1.28)	0.62 (0.30–1.29)	-
*Female X* *Welfare State*						
Female X SOU	**2.42 (1.86–3.15)**	**2.43 (1.86–3.16)**	**2.42 (1.85–3.15)**	**2.43 (1.86–3.17)**	**2.42 (1.86–3.15)**	**2.38 (1.84–3.08)**
Female X CON	-	1.42 (0.81–2.50)	**2.28 (1.28–4.08)**	1.59 (0.88–2.87)	1.56 (0.79–3.07)	1.49 (0.83–2.67)
Female X LIB	**2.20 (1.27–3.80)**	**-**	**3.06 (1.56–6.00)**	**1.93 (1.11–3.38)**	**2.03 (1.15–3.60)**	**2.13 (1.24–3.66)**
Female X SD	1.97 (0.62–6.24)	1.25 (0.42–3.76)	-	1.71 (0.55–5.30)	1.77 (0.53–5.92)	1.80 (0.59–5.45)
Female X SA	**0.22 (0.09–0.50)**	**0.27 (0.12–0.61)**	**0.14 (0.05–0.38)**	**-**	**0.24 (0.08–0.70)**	**0.25 (0.11–0.59)**
Female X EE	0.79 (0.50–1.23)	0.83 (0.54–1.26)	0.61 (0.33–1.15)	0.80 (0.51–1.25)	-	0.92 (0.59–1.45)
Female X LA	1.19 (0.61–2.32)	1.67 (0.88–3.17)	0.88 (0.40–1.93)	1.35 (0.69–2.65)	1.31 (0.64–2.68)	-
Intercept	2.391	3.063	1.995	2.583	2.462	1.960
Variance	0.094	0.156	0.153	0.160	0.191	0.134
−2logL	−18,202.482	−17,893.987	−18,481.133	−17,705.833	−18,240.581	−8,093.586
*N*	86,465	86,490	88,086	75,955	86,359	45,337

*Notes*: Models 1 to 6 exclude Conservative, Liberal, Social Democratic, Southeast Asia, Eastern European, and Latin American welfare regimes, respectively; All models adjust for age, educational attainment, marital status, GDP per capita and GDP annual growth rate (not shown); ORs and CIs in bold are significant at *p* < 0.05.
